# Development and Evaluation of a Novel Diammonium Glycyrrhizinate Phytosome for Nasal Vaccination

**DOI:** 10.3390/pharmaceutics14102000

**Published:** 2022-09-21

**Authors:** Xiaojin Chen, Xudong Fan, Fanzhu Li

**Affiliations:** 1 College of Pharmaceutical Sciences, Zhejiang Chinese Medical University, Hangzhou 310053, China; 2 Department of Pharmacy, Hangzhou Children’s Hospital, Hangzhou 310014, China

**Keywords:** diammonium glycyrrhizinate, phytosome, optimization, nasal delivery, adjuvants

## Abstract

The objective of the present research was to formulate diammonium glycyrrhizinate (DG) into phytosomes (DG-P) to induce nasal immune responses and enhance absorption. Plackett- Burman design was used for process optimization, incorporating specific formulation and process variables to obtain the optimal parameters. Fourier transform infrared spectroscopy (FTIR), X-ray power diffraction (P-XRD), and transmission electron microscopy (TEM) were used for characterization. The adjuvant activity of the DG-P was evaluated by using bone marrow dendritic cells. In vitro nasal mucosal permeation and in situ nasal perfusion were also investigated to evaluate nasal absorption. The DG phytosomes were in the size range of 20~30 nm and zeta-potential range of −30~−40 mV. DG-P demonstrated 4.2-fold increased solubility in *n*-octanol. Coculturing bone marrow dendritic cells with DG-P led to enhanced dendritic cell maturation. Apparent permeability coefficient of the phytosomal formulation was almost four times higher than that of free DG determined by ex vivo permeation studies on excised porcine mucosa. In situ nasal perfusion studies in rats demonstrated that the nasal absorption of DG-P was significantly higher than that of free DG. Conclusively, the results confirmed that DG-P have potential for use as an adjuvant for nasal vaccine.

## 1. Introduction

Vaccination is considered the most powerful and successful method for preventing and controlling infectious diseases. Currently, injection is the most commonly used method of vaccination administration. However, the majority of pathogens invade mucosal surfaces, such as those in the respiratory tract. Therefore, the delivery of vaccines through mucosal surfaces has the potential to stimulate both mucosal and systemic immune responses, thus encountering the infection at the site of pathogen entry [1,2]. Nasal administration of vaccines is optimal for mass vaccination in times of a pandemic, as well as a simple, convenient and needle-free method for vaccinating children [3]. Intranasal vaccination against influenza has been used for many years to prevent this infection. Recently, various nasally administrated COVID-19 vaccines have been under intensive investigation and have been demonstrated to be potent in inducing both mucosal and systemic immune responses [4].

However, very few mucosal vaccines have been approved for human use due to various challenges associated with mucosal delivery, such as poor immunogenicity. Many efforts have been made to enhance the mucosal immunity, such as targeting to the M cells, exploiting suitable adjuvants, etc. Due to the lack of mucus secretion and high activity of endocytosis, M cells could capture antigens in the mucosal lumen efficiently and rapidly, acting as a functional equivalent of lymphatics for mucosal lymphoid tissues [5]. Then, the dendritic cells (DCs) housed in the pockets of M cells directly take up the antigens transported by M cells, and rapidly present the antigens to B and T cells adjacent to the basolateral compartment of M cells, inducing antigen-specific immune responses. Thus, M cell ligands could be used as novel and effective mucosal vaccine targets to increase antigen uptake and presentation. However, this vaccination design is limited by the availability and feasibility of the delivery methods, as well as the suitable M cell models for formulation development [6]. Adjuvants are crucial for the development of efficient mucosal vaccines [7,8,9]. Alum is a poor inducer of mucosal immunity, and concerns about its toxicity in infant and pediatric populations who are exposed to amounts of alum much higher than the dose limit are warranted [10]. Therefore, potential adjuvants derived from plants or herbs have been extensively investigated. Saponins (e.g., QS-21) or saponin-based particulates such as immunostimulatory complexes (ISCOMs) or ISCOMATRIX are promising for vaccine development [11,12,13]. Currently, QS-21 is licensed for human use in malaria and herpes zoster vaccines. In addition to QS-21, several saponins from herbs have been investigated, such as ginsenoside Rg1 and astragaloside VII, which have demonstrated potential as natural sources of vaccine adjuvants [14,15,16]. Licorice, the roots of Glycyrrhiza, has been historically used as medicine in China, India, Egypt, and Greece. It is used to treat many diseases, such as peptic ulcers, hepatitis, epilepsy, fever, respiratory diseases, and skin problems [17]. Modern pharmacological studies have demonstrated that licorice has a variety of activities including anti-inflammatory, antimicrobial, antiviral, antiasthma, anticancer, and immunomodulatory as well as hepatoprotective, gastroprotective, and cardioprotective effects [18,19]. The root extract mainly contains triterpenoid saponins with glycyrrhizin as the main constituent. Glycyrrhizin is the most potent component in licorice, and it is found naturally as a mixture of the potassium and calcium salts of glycyrrhizic acid. Diammonium glycyrrhizinate (DG) is an ammonium salt of glycyrrhizic acid that is used medically in China as a third generation of glycyrrhizic acid [20]. Saponins isolated from licorice have been reported to stimulate high levels of humoral and cellular immunity [21,22].

To reduce toxicity and haemolysis, saponins have been mixed with cholesterol and phospholipid to comprise the adjuvant named ISCOMATRIX^®^. ISCOMATRIX^®^ forms nanoparticles with a diameter of ~40 nm. Novavax developed a saponin-based matrix particle (Matrix-M1, 40 nm) as an adjuvant [23]. The COVID-19 vaccine, mixed with Matrix-M1, demonstrated 86.3% protection against the B.1.1.7 (Alpha) variant and 96.4% protection against non-B.1.1.7 variants after a two-dose regimen during phase III clinical trials, and the reactogenicity was generally mild and transient [24]. Nanoparticles have been extensively used as adjuvants and antigen delivery vehicles, inducing both cellular and humoral immunity, providing long-lasting immunogenic memory [25]. Saponin-based nanometer adjuvants are effective at both antigen delivery and immune-stimulation, and considered as an integrated adjuvant system. Saponin-based nanoscale adjuvants not only enforce CD4 + T cell mediated immunity, but also induce CD8+ T cell activation, which have been developed for therapeutic vaccines such as HPV, HCV, or cancer, as well as prophylactic vaccines as influenza or malaria [13,26,27].

In this study, we developed a saponin-phospholipid complex to enhance the immune response and reduce toxicity. Drug-phospholipid complexes, known as phytosomes and pharmacosomes corresponding to herbal and conventional dosage forms, show physicochemical characteristics different from liposomes [28]. Due to covalent or hydrogen bonding interaction with phospholipids, these drug-phospholipid complexes have higher drug loading, greater stability, and lower drug leakage than liposomes [29]. Drug-phospholipid complexes have demonstrated promising results regarding their oral absorption and percutaneous permeation [30,31]. However, the nasal absorption of phytosomes has not yet been investigated.

Therefore, we developed DG phytosomes (DG-P) as a vaccine adjuvant, and their immune activity and nasal absorption and permeation were investigated.

## 2. Materials and Methods

### 2.1. Materials

Diammonium glycyrrhizin (purity 99%) was purchased from Xi’an Puruisi Bioengineering Co., Ltd. (Xi’an, China) and phospholipids with phosphatidylcholine contents of approximately 90% (*w*/*w*, S90) and 80% (*w*/*w*, S80) were purchased from Taiwei Pharmaceutical Ltd. (Shanghai, China). A Cell Counting Kit-8 was obtained from Biosharp (-Hefei, China), and a granulocyte macrophage colony stimulatory factor (GM-CSF) was supplied by GenScript (Piscataway, NJ, USA). Fluorochrome-labelled anti-mouse monoclonal antibodies including APC-CD11c, FITC-CD80, and PE-CD86, were purchased from Proteintech (Rosemont, IL, USA).

Six- to eight-week-old C57BL/6 mice, and SD rats weighing 200–250 g, were obtained from the Zhejiang Center of Laboratory Animals (ZJCLA, Hangzhou, China). All animal protocols were approved by the Institution of Animal Ethical and Welfare Committee of ZJCLA (Approval Code: ZJCLA-IACUC-20020064, Approval Date: 31 October 2021).

### 2.2. Preparation of DG-P

The DG-phospholipid complex was prepared using the solvent evaporation technique as described in the literature [32,33,34]. Phospholipids (90% or 80% purity) and DG (at a ratio of 1.4 or 0.7, *w*/*w*) were dissolved in tetrahydrofuran, and the mixture was stirred with a magnetic agitator at 400 or 600 rpm. The complexation temperature was controlled at 40 °C or 60 °C using a water bath for 3 h or 5 h. The suspension was filtered through a 0.22 μm pore size organic membrane to remove excess DG. Then, the clear solution was evaporated under vacuum at 30 °C for 1 h, and the dried residue was placed in a desiccator overnight and then stored in a glass bottle. The solid DG-phospholipid complexes were dispersed in phosphate-buffered saline (PBS, pH 6.5) to form homogeneous phytosomal dispersions [35,36].

### 2.3. Plackett- Burman (PB) Design

To reduce the number of trials and explore the formulation factors and process variables that affect the critical quality attribute (CQA) values of the phytosomes, a PB design was adopted with six factors and twelve experiments. The parameters studied were: complexation time (X_1_), complexation temperature (X_2_), phospholipid concentration (X_3_), agitation speed (X_4_), lipid/drug ratio (X_5_, *w*/*w*), and phosphatidylcholine purity (X_6_). Each of these factors was evaluated at two levels, and the parameters and relevant levels are presented in Table 1. The selected responses were yield (Y_1_), drug loading (Y_2_), particle size (Y_3_), polydispersity index (PDI) (Y_4_) and zeta potential (Y_5_). Minitab 19.0 was used for the generation and randomization of the PB design (Table 2) as well as the subsequent statistical analysis. The statistical significance of each parameter was determined by ANOVA of the linear model based on the *p* value (<0.05).

The linear models were obtained as follows by regression analysis for each individual response:

Y = β_0_ + β_1_X_1_ + β_2_X_2_ + β_3_X_3_ + β_4_X_4_ + β_5_X_5_ + β_6_X_6_ (1)

where Y is the response, X_i_ refers to the factor under design, β_0_ is a constant, and β_i_ is the coefficient of each factor.

### 2.4. Optimization of the DG-P

The formulation of the DG-P considered the optimization of the desirability of the main responses based on maximum yield% (Y_1_), maximum drug loading (Y_2_), minimum particle size (Y_3_) and minimum PDI (Y_4_). The factor screening results demonstrated that the zeta-potentials (Y_5_ values) of the different formulations of DG-P ranged from −30 mV to −40 mV, which were appropriate for particle stability. As a result, the zeta- potential response was not included in the optimization process.

### 2.5. The Yield of the DG-Phospholipid Complex

Both the phospholipid complexes and phospholipids easily dissolved in tetrahydrofuran, but DG was practically insoluble in this solvent. The free DG was separated by filtration through a 0.22 μm membrane, dried in a desiccator overnight, and then weighed. The yields of DG-phospholipid complexes “present as a complex” was calculated using the following equation [37]:

The Yield (%) = (W1 − W2)/W1 × 100%(2)

where W1 is the amount of DG added during the synthesis of the complex and W2 is the amount of free DG not included in the complex.

### 2.6. Determination of Drug-Loading Content in the Phospholipid Complex

The determination of diammonium glycyrrhizin content was performed on an HPLC system (HP 1200 series, Agilent, Santa Clara, CA, USA). Separation was carried out on a Zorbax SB C_18_ column (250 mm × 4.6 mm, 5 μm) with a column temperature of 30 °C. The mobile phase consisted of a mixture of acetonitrile:0.01 M H_3_PO_4_ (40:60). The flow rate was 1.0 mL/min, and the detection wavelength was 250 nm. Approximately 10 mg of each DG-phospholipid complex was dissolved in 10 mL methanol, sonicated for 10 min, and diluted to 100 mL with solvent A (acetonitrile:distilled water = 60:40, *v*/*v*), and 20 μL was injected into the HPLC system [38]. The calibration curve was obtained by linear regression of the peak area versus concentrations in the range of 5 μg/mL to 100 μg/mL (*r*^2^ = 0.9998). Drug-loading content was calculated using the following equation [34]:

Drug-loading content% = (amount of DG in theDG-P)/amount of DG-P × 100%(3)

### 2.7. Particle Sizes and Zeta Potential of the DG-P

Approximately 10 mg of the DG-phospholipid complexes were dispersed in 10 mL PBS (pH 6.5) to form a phytosomal suspension. The mean particle sizes, PDIs, and surface zeta potentials of the DG-P were analyzed using a zetasizer (ZS90, Malvern Instrument, Inc., Worcestershire, UK). All measurements were conducted in triplicate at 25 °C. The average value of each was used.

### 2.8. Characterization of the DG-P

#### 2.8.1. Transmission Electron Microscopy (TEM)

Morphological examination of the optimized formulation (FR2) was performed using a transmission electron microscope (HT7700, Hitachi Ltd., Tokyo, Japan). One drop of the phytosomal dispersion was placed on a carbon-coated copper grid and stained with phosphotungstic acid (2%, *w*/*v*), and excess stain was removed with filter paper. The grid was air dried and then viewed by TEM.

#### 2.8.2. Fourier Transform Infrared Spectroscopy (FTIR)

The infrared spectra of pure DG, phospholipids and the DG-phospholipid complexes were analysed using a Nicolet IS50 spectrometer (Thermo Fisher Scientific, Waltham, MA, USA). The conventional method using KBr pellets was carried out in the wavelength range of 4000 to 400 cm^−1^.

#### 2.8.3. Powder X-ray Diffraction (PXRD)

The PXRD patterns of pure DG, phospholipids and the DG-phospholipid complexes were obtained using an X-ray diffractometer (Shimadzu XRD-7000, Kyoto, Japan). The instrument was operated at 40 kV and 30 mA. Samples were scanned with a diffraction angle range from 4° to 60° 2θ at a scan rate of 0.04°2θ/min.

### 2.9. Solubility of the DG in Water and n-Octanol

Excess DG or DG-phospholipid complex was added to 5 mL of *n*-octanol or water in sealed glass containers at 25 °C. The liquid was agitated for 24 h, followed by centrifugation at 4000 rpm for 15 min to remove excessive residues. The supernatant was filtered through a 0.45 μm membrane. One milliliter of filtrate was diluted with an appropriate amount of methanol and analysed using HPLC.

### 2.10. Haemolytic Activity of DG-P

The haemolytic activity of DG and the DG-P was determined, as previously described [39]. Human red blood cells (RBCs) were collected by centrifugation and resuspended in saline solution. A 1 mL volume of the cell suspension was mixed with 1 mL of either 0.005%, 0.01%, 0.05%, 0.075% or 0.1% (*w*/*v*) solution of DG or DG-P. Double distilled water was used as the positive control (100% haemolysis) and saline was used as the negative control. After incubation for 1 h at 37 °C, each tube was centrifuged for 5 min at 2000 rpm. The absorbance of the obtained supernatant was measured using a UV spectrophotometer at 415 nm.

### 2.11. Bone Marrow Dendritic Cells (BMDCs) Culture, Viability, and Activation

BMDCs have been used as a primary in vitro model to study DC biology since 1992 [40]. DCs are potent, efficient, and professional antigen-presenting cells (APCs) during immune responses, linking the innate immune response to an antigen-specific adaptive response. Therefore, BMDCs are extensively used to evaluate various vaccines and adjuvants, from therapeutic to prophylactic, from subcutaneous injection to intranasal instillation [41,42,43,44]. BMDCs were obtained from the bone marrow of C57BL/6 mice and cultured in RPMI 1640 medium containing 10% foetal bovine serum (FBS), 1% penicillin, 1% streptomycin, and 25 ng/mL GM-CSF at 37 °C, 5% CO_2_. Half of the medium was replaced with fresh medium on day 3 and day 5. On day 7, BMDCs were collected for further in vitro experiments [45].

BMDCs viability was measured by CCK-8 assay. BMDCs were separately seeded in 96-well plates at a density of 5000 cells/well and incubated with 100 μL of different concentrations (0.01, 0.1, 1, 10, 100 μg/mL) of DG-P or free DG. After incubation for 24 h, the supernatant was removed, and 100 μL of 10% CCK-8 solution was added. After 2 h of incubation, the absorbance of each well was measured at 450 nm using a plate reader.

To further determine whether the DG-P could act as an adjuvant to induce the activation of DCs in vitro, BMDCs were incubated with free DG or DG-P for 24 h, and harvested and stained with antibodies against CD11c, CD80, and CD86 for 30 min at 4 °C. After washing with PBS, the expression of CD11c, CD80, and CD86 on DCs was measured by a flow cytometer (BD Biosciences, San Jose, CA, USA).

### 2.12. Ex Vivo Permeation Study of the DG-P

Permeation experiments were carried out on modified Franz diffusion cells using excised porcine nasal mucosa obtained from a local slaughterhouse [46,47]. DG solution or DG-loaded phytosomal dispersion (2.0 mg/mL in PBS) was placed on the membrane surface, and the donor compartment was sealed using Parafilm to achieve occlusive conditions. The receiver compartment was filled with PBS (pH 6.5) remaining at 37 ± 1 °C with constant stirring to ensure homogeneity. At the appropriate time intervals, 1 mL of the receptor phase was withdrawn and the compartment was refilled with the same volume of prewarmed PBS. The concentration of released DG was analysed via HPLC. The cumulative amount of drug permeated per unit surface area (μg/cm^2^) versus time (h) was plotted. The flux (*Jss*, μg/cm^2^/h) of the drug was calculated according to Equation (4):

(4)   Jss=dQ/dt×1/A 

where *Q* is the accumulative amount of drug in receiver compartment at time *t*, *dQ/dt* is the linear regression of the steady-state portion of the plot (μg/h), and A is the area of the effective surface area (cm^2^). The apparent permeability coefficient (*Papp*, cm/h) was obtained using Equation (5) [48]:

*Papp* = *Jss*/*C*_0_ (5)

where *C*_0_ is the initial concentration in the donor compartment (μg/mL).

### 2.13. In Situ Nasal Perfusion

In situ nasal absorption experiments were carried out following the reported methods with slight modification [49,50]. Each rat was first anaesthetized by the intraperitoneal (IP) administration of an anesthetic mixture of Zoletil 50 (32 mg/kg of body weight) and dexmedetomidine (0.1 mg/kg of body weight) and affixed supine on a board. Then, the trachea was cut and cannulated with a polyethylene tube to aid breathing. Another tube was inserted through the oesophagus towards the posterior of the nasal cavity, and the nasopalatine was blocked with an adhesive agent to prevent the loss of perfusion into the oral cavity. The tube from the nasopalatine was inserted into a reservoir containing 10 mL of DG solution or phytosomal dispersion, and the solution was circulated with a peristaltic pump from the drug reservoir through the nasal cavity and out of the nostrils back into the reservoir at a rate of 2.0 mL/min. Two hundred microlitres of perfusate was withdrawn and replaced with an equal volume of drug solution. The experiment lasted for 120 min. The concentrations of DG were determined by HPLC.

## 3. Results and Discussion

### 3.1. Experimental Design

Solvent evaporation is the most common and easiest method to prepare phospholipid complexes. Factors such as temperature, complexation time, and lipid/drug ratio as well as their concentrations may affect the desired performance of the phospholipid complex in terms of drug loading, yield, and size. In the literature, the phosphatidylcholine content has been reported to vary from 60% to 95%, which might have influenced the size and stability of the phytosomes [51]. The value of each parameter was based on prior knowledge and the literature.

The results of the PB design are presented in Table 3, and ANOVA of the five responses along with the *p*-values are listed in Table 4. A positive sign associated with the coefficient indicated a positive correlation between the studied variables and the response, while a negative sign indicated a negative correlation. The results revealed that the complexation temperature, phospholipid concentration, and lipid/drug ratio highly influenced the critical qualities of the phytosomes. The purity grade of phospholipids had no effect on the particle size or zeta potential of the phytosomes, which might be because the phospholipid purity we used was above 80%. The complexation time and agitation speed had no effect on the experimental results based on the two levels.

#### 3.1.1. Influences of the Independent Variables on Yield

The yields (%) of the 12 batches demonstrated a wide range from 54.08% (F1) to 99.56% (F5). According to the regression analysis of the responses, the complexation temperature, lipid concentration, and lipid/drug ratio had a statistically significant influence on yield. The positive signs before the coefficient of complexation temperature (8.02) and lipid concentration (6.10) indicated a positive effect on yield, while the negative sign associated with the lipid/drug ratio (−5.79) revealed its inverse relationship between with yield. When the temperature was 40 °C, the mixture of lipids and DG required more than 5 h to completely interact, while when the temperature was raised to 60 °C, the mixture became clear after 1 h. Thus, the complexation temperature was more important than the complexation time. The positive effect of lipid concentration might be due to the increased density of phospholipid chains interacting with the COOH- and OH- groups of DG. The effect of the phospholipid: drug ratio on yield has been demonstrated in different studies. Telange et al., prepared apigenin-phospholipid phytosomes and the yield was higher when the phospholipid: apigenin ratio was 2:1 (*w*/*w*, equivalent to a molar ratio of 1:1.4) rather than 3:1 and 1:1 [52]. Qin et al., prepared a bergenin-phospholipid complex by a series of ratios (0.4, 0.57, 0.8, 0.9, and 1.2; drug: phospholipid, *w*/*w*), and the yield increased with the proportion of phospholipids [37]; when the ratio was more than 1.2, the yield decreased. Dora et al., prepared gemcitabine phospholipid complexes by solvent evaporation with different molar ratios (2:1, 1:1, 1:2 and 1:4) of gemcitabine: phospholipids [53]. The maximum yield and drug loading were obtained with a 1:1 drug: phospholipid ratio. As a result, the optimum ratio of drug: phospholipids varies according to the molecular structure of the drug.

#### 3.1.2. Influence of the Independent Variables on Drug Loading

Drug loading for the tested batches ranged from 18.69% (F1) to 61.55% (F2). Regression analysis demonstrated that the lipid/drug ratio had a significant effect on drug loading; the higher the percentage of phospholipids was, the lower the drug loading in the complexes, which was consistent with the literature [37,54]. This effect could be due to the drug content in the phytosomes being determined by calculating the proportion of drug in the phospholipid complex, which is affected by the initial lipid/drug input ratio.

#### 3.1.3. Influence of the Independent Variables on Particle Size

For the twelve batches examined, the observed particle sizes ranged from 19.09 nm to 171.93 nm. The coefficient values of the complexation temperature and lipid/drug ratio were found to be significant. The negative sign before the linear coefficient of complexation temperature (−17.27) indicated its negative effect on particle size. This result could be explained by an increase in temperature leading to a strong complexation and tight integration between the drug and phospholipids, causing the formation of particles with smaller diameters. The positive sign before the linear coefficient of the lipid/drug ratio (37.55) indicated its positive effect on particle size, i.e., as the proportion of phospholipids increased, the particle size increased. Freag et al., prepared diosmin phytosomal carriers at different drug:lipid molar ratios (1:1, 1:2, 1:3, 1:4), and the minimum particle size was found with a drug:lipid ratio of 1:2 [54]. When the ratio was increased to 1:4, the phytosomes were larger. Song et al., prepared breviscapine phospholipid complexes at different drug-lipid mass ratios (1:1, 1:2, 1:3), and the particle size tended to increase with increasing lipid content [55]. One possible explanation for this result was that excessive amounts of phospholipid molecules contributed to aggregation, leading to a larger size.

#### 3.1.4. Influence of the Independent Variables on Zeta Potential

The zeta potentials of the tested formulations ranged from −29.3 (F12) to −40.7(F9). Phytosomes with negative zeta potentials have been reported by many studies, which could be attributed to the negatively charged phospholipids in aqueous environments. Stability is a general limitation of lipid vesicles. Additionally, a large proportion of phospholipids would induce a highly negative zeta potential. A high surface charge (≥25 mV) leading to particle repulsion ensures the stability of phytosomes and protects them from flocculation [56].

### 3.2. Optimization of the Phytosomes

Based on the PB design, the main significant factors and influencing responses were identified. The responses of the yield, particle size, and zeta potential were affected by the tested variables of complexation temperature, lipid concentration, and lipid/drug ratio. High yield and maximum drug loading are highly critical quality attributes for phytosomes, while a smaller particle size, lower polydispersity index and modest zeta potential value suggests good physical stability. According to the PB design, increasing temperature and lipid concentration could result in high yield and a small particle size. However, the lipid/drug ratio had the opposite effect on yield and particle size, as a high lipid/drug ratio might lead to a high yield and large particle size. As a result, the optimum values of the formulation factors were determined as follows: complexation at 60 °C for 3 h, phospholipids (90%) concentration of 10 mg/mL, and agitation speed of 800 rpm. The optimum lipid/drug ratio ranged from 1.2 (minimum particle size below 100 nm; minimum PDI below 0.2) to 0.7 (minimum particle size below 50 nm; minimum PDI below 0.3). Optimization of the lipid/drug ratio was then carried out, and the results are shown in Table 5. Accordingly, the yields of these four formulations were higher than 99%, the particle sizes were smaller than 100 nm, and the PDI values were below 0.3. High drug loadings in the optimized formulations were obtained ranging from 45.19% to 56.82% due to the high yield and drug/phospholipid ratio. Demana et al., investigated the pseudo-ternary phase diagrams of the formation of ISCOM matrics by the lipid-film method, and the results demonstrated that ISCOM matrics could be found in systems containing 40% phospholipid, 40% Quil-A, and 20% cholesterol, indicating that a 1:1 mass ratio of saponin to phospholipid was preferred [57]. According to the drug loading, the mass ratio of DG and phospholipid in the optimization formulations were all close to 1:1, especially FR2. Considering the convenience of operation, the lipid/drug ratio of 1.0 was adopted for the following experiments.

### 3.3. TEM

**Figure 1:**
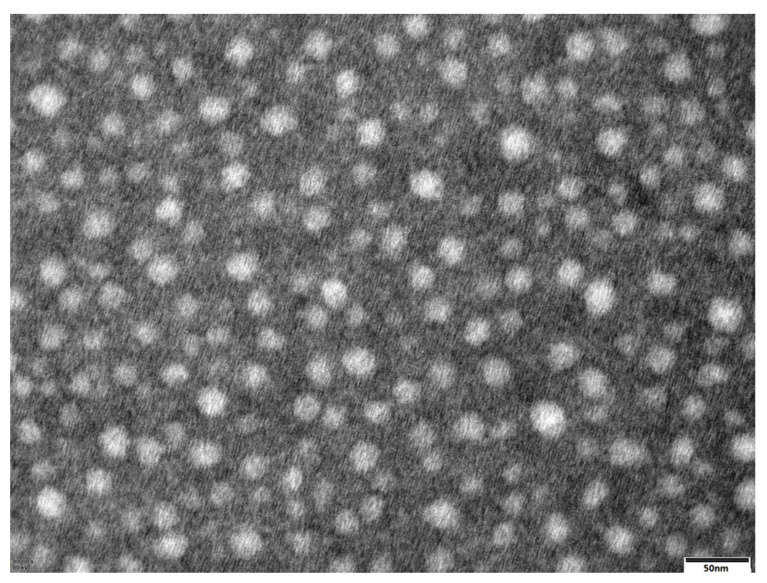
TEM photographs of DG phytosomes (×40,000).

TEM images of the optimized formulation demonstrated that the morphology of the phytosomes exhibited a well distributed spherical particle shape, as shown in Figure 1. The TEM results demonstrated that the diameters of the DG-P were approximately 20 nm.

### 3.4. FTIR

**Figure 2:**
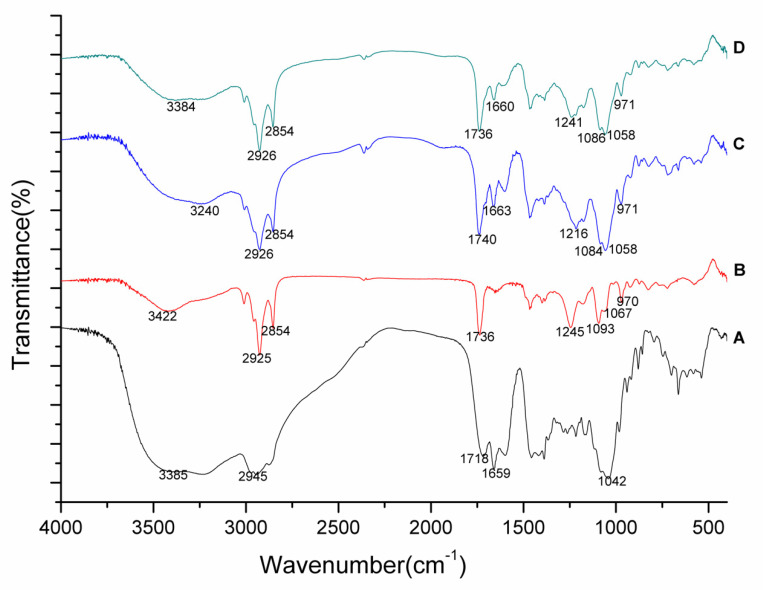
FTIR spectra of DG (A), phospholipid (B), DG-P (C), and physical mixture (D).

The FTIR spectra of DG, phospholipids, the DG-P, and a physical mixture of DG and phospholipids (PM) are shown in Figure 2. The spectrum of DG revealed characteristic peaks at 3385 cm^−1^ (O-H stretching), 2945 cm^−1^ (saturated alkyl C-H stretching), 1718 cm^−1^ (COOH stretching), 1659 cm^−1^ (C=C stretching), and 1042 cm^−1^ (C-O-C stretching).These observations were in agreement with those reported earlier [58,59]. The phospholipids spectrum exhibited characteristic peaks at 2925 cm^−1^ (C-H stretching), 2854 cm^−1^ (C-H stretching), 1736 cm^−1^ (C=O stretching), 1245 cm^−1^ (P=O stretching), 1093 cm^−1^ (P–O symmetric stretching), 1067 cm^−1^ (C–O–P stretching), and 970 cm^−1^ (N^+^(CH_3_)^3^ stretching). The characteristic peaks at 3385 cm^−1^(O-H stretching) of DG shifted to a lower wavelength (3240 cm^−1^) in the sample of DG-P, which also demonstrated a decrease in the intensity. There was a clear shift of the characteristic peak of phospholipids at 1245 cm^−1^ (P=O stretching) to 1216 cm^−1^ in the sample of DG-P, accompanied by shifts of the peaks at 1093 cm^−1^ (P–O symmetric stretching), 1067 cm^−1^ (C–O–P stretching), and 1736 cm^−1^ (C=O stretching) to lower wavenumbers (1084 cm^−1^, 1058 cm^−1^, and 1740 cm^−1^, respectively). In addition, the absorption peak of DG at 1718 cm^−1^ (COOH stretching) disappeared in the sample of DG-P, while the N^+^(CH_3_)^3^ vibration of phospholipids at 970 cm^−1^ shifted to 971 cm^−1^. These observations suggest that the hydroxyl and carboxyl groups in DG interacted with the phosphate group and/or N atoms of the phospholipids during phytosome formation. These interactions could be noncovalent bonds, such as hydrogen bonds and electrostatic interactions, rather than interactions that formed via the generation of a new compound. On the other hand, the strong C-H stretching absorptions at 2926 cm^−1^ and 2854 cm^−1^ from the phospholipids were retained in the phytosome sample, indicating that the long fatty acid chains did not associate during the formation of the phytosomes.

### 3.5. PXRD

**Figure 3:**
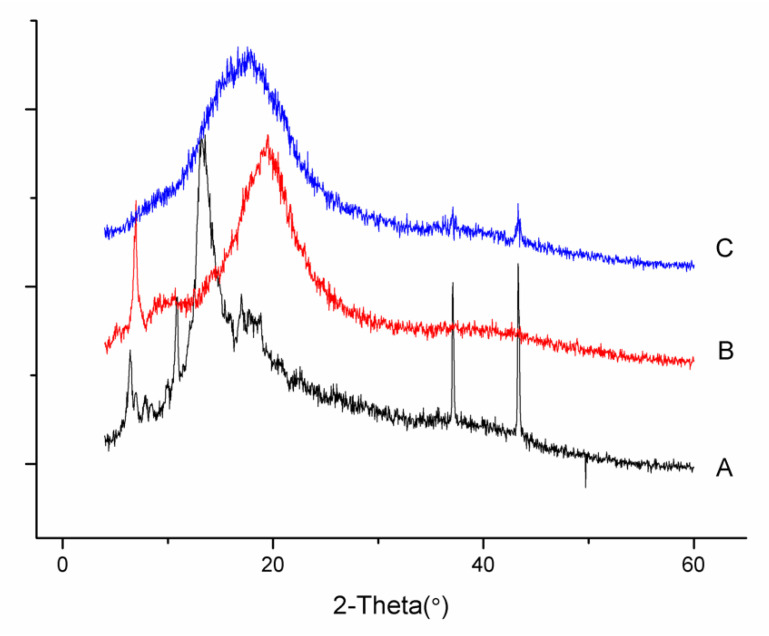
PXRD spectra of DG (A), phospholipid (B), and DG-P (C).

The X-ray diffractograms of free DG, phospholipids, and the DG-P are shown in Figure 3. Free DG displayed intense, sharp peaks, indicating its crystalline nature. The diffractogram of the phospholipids exhibited an amorphous state. For the DG-P, the typical crystal peaks of DG disappeared, and the diffractogram was similar to that of the phospholipids. However, some crystalline drug signals still appeared in DG-P G around 37.08 and 43.32° 2θ but tended to weaken, indicting the DG is partially converted to the amorphous form or is more likely embedded in the phospholipids’ matrix [38,60,61].

### 3.6. Solubility Analysis

Pure DG and the DG-P had high solubility in water (greater than 100 mg/mL), indicating their rather hydrophilic nature. The solubilities of DG and the DG-P in *n*-octanol were 522.05 ± 84.52 μg/mL and 2157.06 ± 50.73 μg/mL, respectively. The phospholipid complexes increased the *n*-octanol solubility of DG to be 4.13 times higher than that of the pure drug. These results indicated that phytosomes could enhance the lipophilicity of the drug. The polar head groups of the phospholipids interacted with DG and the long hydrocarbon tail enveloped the polar groups of the drug, such as hydroxyl groups and carboxyl groups [62]; thus, the lipophilicity of DG increased, possibly enhancing its permeability through the intestinal epithelium.

### 3.7. Haemolytic Activity

**Figure 4:**
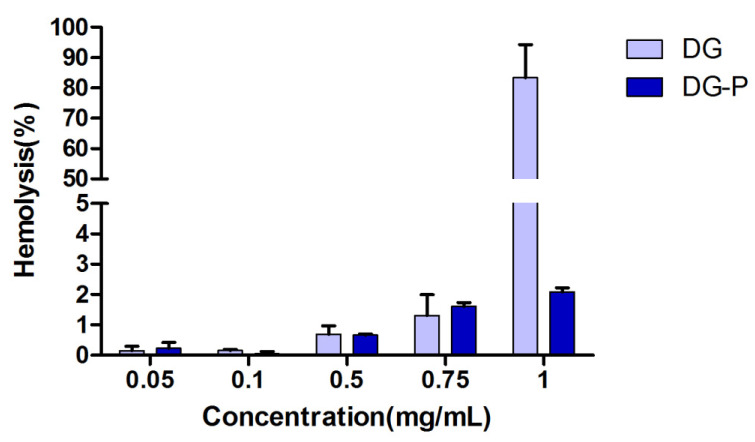
Haemolysis induced in RBCs following incubation with free DG and DG-P (*n* = 3). Double distilled water was taken as the positive control (100% haemolysis) and saline as the negative control.

The haemolytic activities of the saponin DG and the DG-P are shown in Figure 4. Haemolytic activity was concentration dependent, and free DG was found to possess much higher haemolytic activity than the DG-P at 0.1% *w/v*. The haemolytic activity of saponins is mainly due to the presence of saccharide side chains and acyl residues on the aglycon [63]. Conjugation of saponins with phospholipids reduces their haemolytic activity, which might be a result of the interaction between the carboxyl groups of DG and the phospholipids. By binding to the phospholipid, the acyl group of DG becomes hidden, and the increasing steric hindrance reduces the ability to bind to the sterols on the cell membrane.

### 3.8. In Vitro Cell Viability

**Figure 5:**
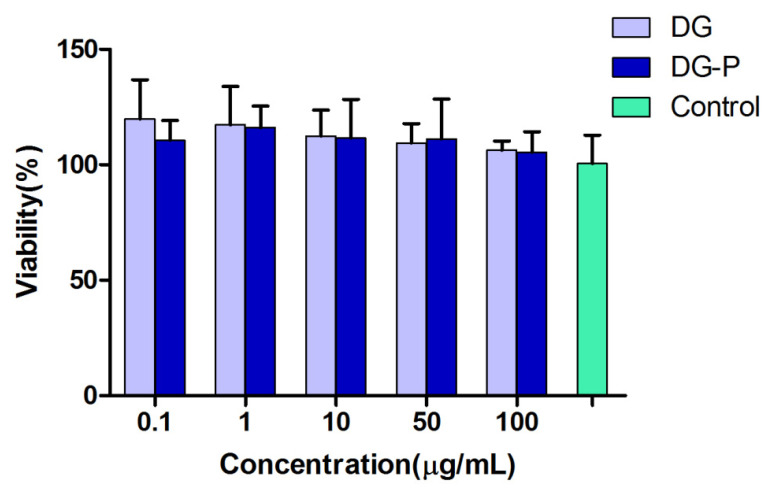
Cell viability of BMDCs incubated for 24 h with DG and DG-P at different concentrations (*n* = 6). No significant toxicity was observed of DG or DG-P.

Safety with antigen-presenting cells (APCs) is crucial for vaccine adjuvants; thus, the toxicity of the DG-P to BMDCs was investigated. As presented in Figure 5, no cytotoxicity was observed for either DG or DG-P compared with the control group, which suggests their potential use in vaccine delivery. Therefore, the subsequent cell experiment was performed at a concentration of 100 μg/mL.

### 3.9. Maturation of BMDCs

**Figure 6:**
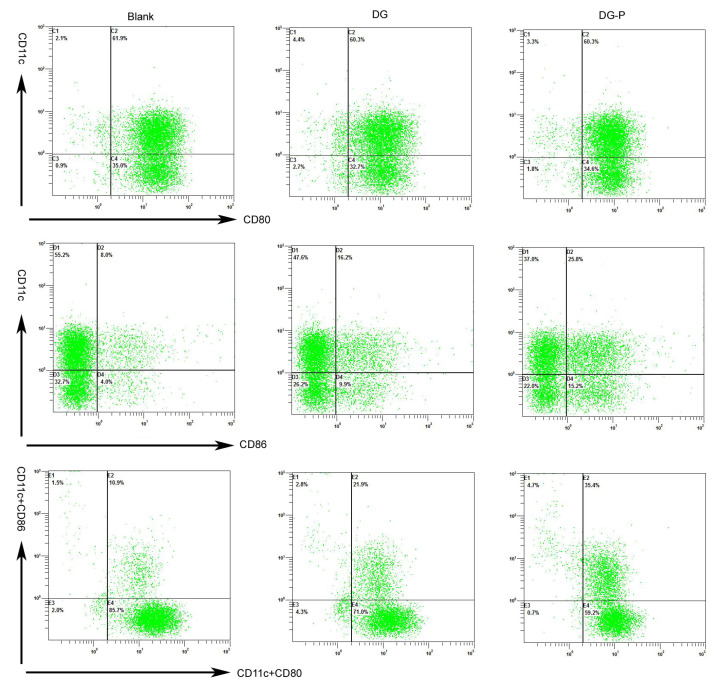
Flow cytometric analysis of CD11c, CD80, and CD86 expression in BMDCs after treatment of blank medium, free DG, and DG-P for 24 h (*n* = 4).

**Figure 7:**
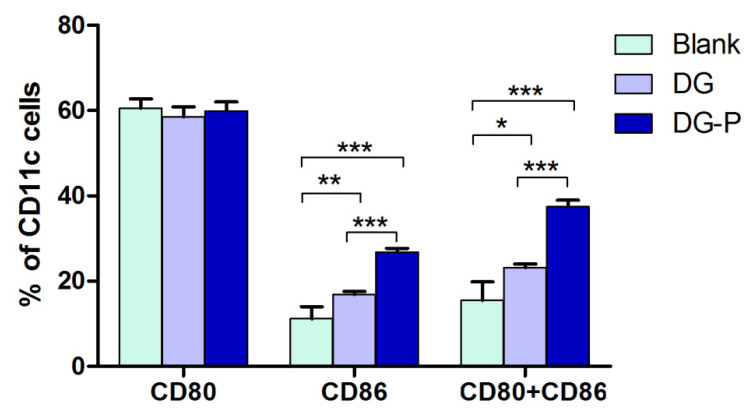
Quantification of CD80 and CD86 expression on BMDCs after activation (*n* = 4). Note: *, ** and *** represent the differences at *p* < 0.05, *p* < 0.01, and *p* < 0.001, respectively.

DCs are the most potent APCs that link the innate immune response to an antigen-specific adaptive response. Mature DCs can present an antigen to T cells to activate them and initiate T-cell immune responses. Therefore, the level of DCs maturation is critical for the immune response. The costimulatory molecules CD80 and CD86 on the DC membrane are important markers of DC maturation. Thus, the expression of CD11c, CD80, and CD86 on BMDCs was analysed by flow cytometry analysis after incubation with free DG and the DG-P. As shown in Figure 6, both DG and the DG-P increased the expression of CD86 compared to the blank control, indicating an immunomodulatory effect of DG. In particular, stimulation with DG-P remarkably improved the expression of costimulator (CD86^+^ CD80^+^), and the average percentage of CD80^+^ CD86^+^ DCs after treatment with the DG-P was 35.4%, compared to 21.9% in the DG group and 10.9% in the blank control (Figure 7). The differences in the activation of DC maturation caused by DG and DG-P administration indicated that the phospholipid complexes (phytosomes) have immunostimulatory properties, which might be attributed to lipid-based nanoparticles. Lipid nanoparticles, such as ISCOMs and liposomes, have been demonstrated to have immunoregulatory effects. Treatment with neither DG nor the DG-P treatment elevated the expression of CD80 compared to that in the untreated groups, which may indicate that DG and DG-P promote CD86 expression and induce Th-2 responses. Montes-Casado et al. [64] reported that treating BMDCs from C57BL/6 mice with Poly(I:C), increased the expression of CD86 but not CD80, which is consistent with our report. These same results were also found by Liang X et al. They evaluated the adjuvant effect of nanoparticles composed of poly(lactic-co-glycolic acid)-polyethylene glycol-polyethylenimine (PLGA-PEG-PEI), and the results demonstrated that the expression of CD86 was enhanced while that of CD80 was not [65].

### 3.10. Ex Vivo Permeation Study of the DG-P

**Figure 8:**
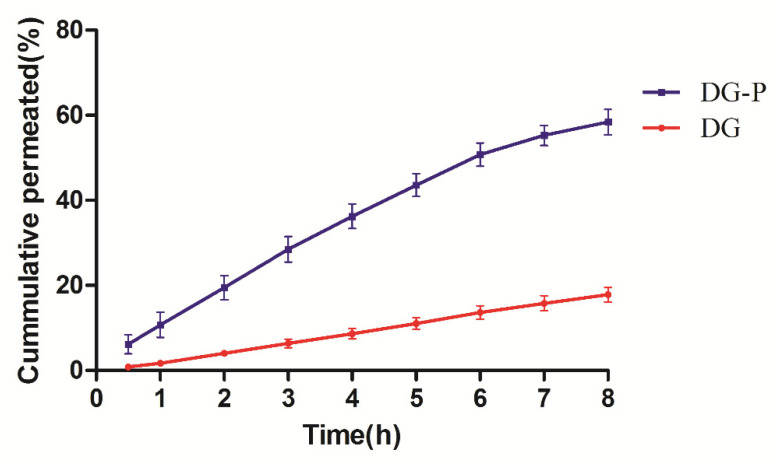
Ex vivo nasal mucosal penetration of DG and DG-P on excised porcine nasal mucosa (*n* = 4).

The nasal mucosal permeation ability of pure DG and the DG-P was presented in Figure 8. Phytosomal formulation significantly enhanced the penetration of DG. The permeability of DG was assessed by calculating the Jss and Papp, which are summarised in Table 6. The permeation rate and amount of DG from the phytosomes were both significantly higher than those from the aqueous solution. The flux of DG from the phytosomes was 114.35 ± 26.54 μg·cm^−2^·h^−1^, which was almost two times higher than that from aqueous solution. In addition, the Papp of DG from the phytosomes was four times higher than that from the aqueous solution. Therefore, the phytosomal system was able to promote the nasal mucosa penetration of water-soluble drugs such as DG. There may be three reasons that can explain the superior permeability of this phytosome system: (a) the lipophilicity of DG increased after its encapsulation into the phytosomes; (b) the phospholipids in the phytosomes facilitated the fusion of the vesicles with the mucosa; and (c) intact vesicles penetrated into and through the mucosa, especially those with small particle sizes.

### 3.11. Absorption Study of DG-P In Vivo

**Figure 9:**
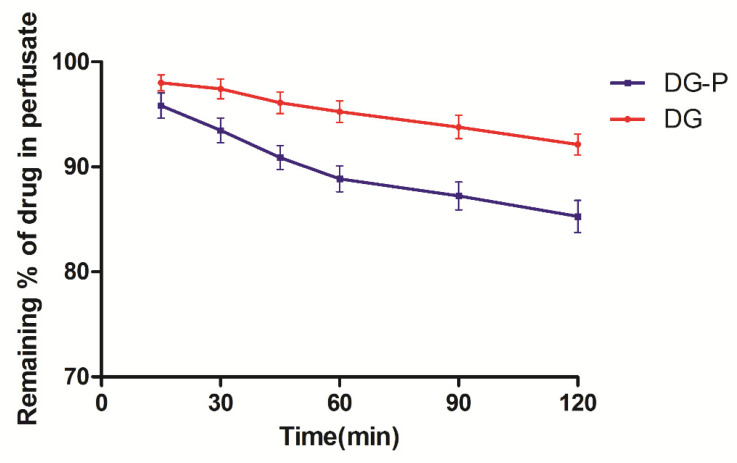
In situ absorption study of DG and DG-P after nasal perfusion on rats (*n* = 6).

Investigating the nasal absorption is critical during screening for a formulation intended for nasal administration. An in situ nasal perfusion experiment was carried out to compare the nasal absorption properties of DG and the DG-P. The absorption profiles were calculated from the drug concentration remaining in the perfusion solution versus time and are shown in Figure 9. DG loaded in phytosomes exhibited better nasal absorption than the pure drug solution. The nasal absorption of the drug demonstrated first-order elimination, and the absorption rate constants for DG and the DG-P were calculated from the slopes of the first-order elimination plot. The nasal absorption rate constants for DG and the DG-P were found to be 7.429 × 10^−2^ h^−1^ and 3.663 × 10^−2^ h^−1^, respectively. There was a significant increase in the absorption rate constant for the phytosome formulation (*p* < 0.01) when compared with the pure drug solution. This enhancement might be attributed to the nanosize of the phytosomes, the increased lipophilicity and the interaction between the phospholipids and cell membrane, which was similar to the ex vivo nasal mucus permeation.

## 4. Conclusions

In this study, a phytosomal formulation of DG was successfully prepared. High yield and drug loading, a small particle size and an appropriate zeta-potential were achieved. The stimulation of BMDCs by the DG-P was significantly enhanced compared with the control and pure DG groups, confirming the immune-stimulatory effect of DG-P. Furthermore, in vitro nasal permeation and in situ nasal absorption studies demonstrated that phytosomes can significantly improve the nasal permeability and absorption compared with free DG. These DG phytosomes may represent a potential platform for nasal vaccination. However, this study mainly investigated the role of DG-P as an adjuvant, and the efficacy of combining with vaccine component remains unknown. Further research of antigen loading must be conducted to continue examining the adjuvant effect of DG-P. In addition, the efficacy of nasal vaccine is influenced by other parameters, including mucosal adhesion, nasal epithelium uptake and lymph node targeting, which should also be carefully investigated in future experiments to understand how DG-P exhibits as a nasal vaccine adjuvant. Moreover, in vivo animal immunization and safety also need to be further carried out. This novel strategy of encapsulating DG into phytosomes provides a potential method for the development of adjuvants for nasal vaccination.

## Figures and Tables

**Table 1:** Factors and levels used in the Plackett-Burman design.

Code	Factors	Levels	
		−1	1
X_1_	Complexation time (h)	3	5
X_2_	Complexation temperature (℃)	40	60
X_3_	Lipid concentration (mg/mL)	5	10
X_4_	RPM	400	800
X_5_	Lipid/drug ratio (*w*/*w*)	0.7	1.4
X_6_	Phosphatidylcholine content	80%	90%

**Table 2:** Composition of Plackett-Burman Design batches.

Formulation Code	X_1_	X_2_	X_3_	X_4_	X_5_	X_6_
F1	3	40	5	800	1.4	0.9
F2	5	40	10	400	0.7	0.8
F3	3	40	5	400	0.7	0.8
F4	5	40	10	800	0.7	0.9
F5	5	60	10	400	1.4	0.9
F6	5	60	5	800	0.7	0.8
F7	5	40	5	400	1.4	0.9
F8	3	60	10	800	0.7	0.9
F9	3	40	10	800	1.4	0.8
F10	3	60	10	400	1.4	0.8
F11	5	60	5	800	1.4	0.8
F12	3	60	5	400	0.7	0.9

**Table 3:** Experimental Responses Results in Plackett–Burman Design.

Formulation Code	Y_1_: Yield (%)	Y_2_: Drug Loading (%)	Y_3_: Average Size (nm)	Y_4_: PDI	Y_5_: Zeta Potential (mV)
F1	54.08	18.69190	171.93	0.237	−34.2
F2	98.13	61.54798	26.39	0.380	−30.2
F3	86.13	52.80669	35.81	0.360	−32.3
F4	98.88	56.68897	20.65	0.251	−31.9
F5	99.56	40.33747	56.17	0.311	−36.9
F6	99.4	56.46473	33.32	0.445	−32.6
F7	72.84	32.93106	128.03	0.230	−34.3
F8	99.33	53.09252	19.09	0.237	−30.8
F9	88.07	35.32267	101.77	0.169	−40.7
F10	98.91	40.60759	83.94	0.194	−35.1
F11	98.05	41.6013	64.40	0.221	−40.1
F12	99.16	57.70004	20.39	0.268	−30.9

**Table 4:** Data of the regression analysis for the responses.

	Yield (%)		Drug Loading (%)		Average Size (nm)		PDI		Zeta Potential (mV)	
	Coefficient	*p* Value	Coefficient	*p* Value	Coefficient	*p* Value	Coefficient	*p* Value	Coefficient	*p* Value
β_0_	91.05	0.000	45.65	0.000	63.49	0.000	0.2752	0.000	−34.167	0.000
Complexation time	3.43	0.170	2.61	0.072	−8.66	0.252	0.0311	0.178	−0.167	0.631
temperature	8.02	0.013	2.65	0.069	−17.27	0.049	0.0041	0.845	−0.233	0.872
Lipid concentration	6.10	0.036	2.28	0.103	−12.16	0.129	−0.0182	0.399	−0.100	0.708
rpm	−1.41	0.540	−2.01	0.141	5.04	0.486	−0.0153	0.476	−0.883	0.144
ratio	−5.79	0.043	−10.73	0.000	37.55	0.002	−0.0482	0.059	−2.717	0.008
purity	−3.74	0.142	−2.41	0.090	5.89	0.419	−0.0196	0.369	1.000	0.112

**Table 5:** Optimization of lipid/drug ratio (mean ± SD, *n* = 3).

Formulation Code	Ratio	Yield (%)	PDI	Particle Size	Drug Loading (%)
FR1	1.2	99.14 ± 0.05	0.21 ± 0.04	28.97 ± 1.12	45.19 ± 6.23
FR2	1.0	99.21 ± 0.09	0.10 ± 0.01	22.42 ± 0.15	48.93 ± 1.28
FR3	0.82	99.34 ± 0.12	0.18 ± 0.01	23.43 ± 0.10	53.41 ± 3.84
FR4	0.7	99.35 ± 0.08	0.16 ± 0.01	26.76 ± 0.24	56.82 ± 1.02

**Table 6:** Ex vivo permeation across excised nasal porcine mucosa of DG and DG-P (mean ± SD, *n* = 4).

	8 h Permeated %	Flux (μg·cm^−2^·h^−1^)	Papp (cm·h^−1^ × 10^−2^)
DG	17.83 ± 3.40	58.43 ± 12.39	2.31 ± 0.49
DG-P	58.40 ± 5.97 ^a^	126.36 ± 6.60 ^a^	7.90 ± 0.41 ^a^

Note: ^a^ representation of the comparison with drug solution at *p* < 0.001.

## Data Availability

The data presented in this study are available on request from the corresponding author. The data are not publicly available due to privacy.
